# Supplementary material for: Economic evaluation of a cluster randomized, non-inferiority trial of differentiated service delivery models of HIV treatment in Zimbabwe
Source: PLOS Glob Public Health. 2023 Mar 13;3(3):e0000493. doi: 10.1371/journal.pgph.0000493 (PMC10021451; doi:10.1371/journal.pgph.0000493)
Supplement: S1 Table — (DOCX) [file pgph.0000493.s001.docx]

**S1 Table.** Baseline characteristics

| Characteristic (n (%)) | 3MF  (n=1,919) | 3MC  (n=1,335) | 6MC  (n=1,546) |
| --- | --- | --- | --- |
| Age (median, IQR) | 45 (38-53) | 47 (41-56) | 45 (38-54) |
| Sex |  |  |  |
| Male | 541 (28) | 351 (26) | 445 (29) |
| Female | 1378 (72) | 984 (74) | 1101 (71) |
| Setting |  |  |  |
| Urban | 387 (20) | 387 (29) | 337 (22) |
| Rural | 1532 (80) | 948 (71) | 1209 (78) |
| Currently employed |  |  |  |
| Yes | 765 (40) | 319 (24) | 800 (52) |
| No | 1148 (60) | 1009 (76) | 741 (48) |
| Unknown | 6 (0.3) | 7 (1) | 5 (0.3) |
| Living >9km from facility |  |  |  |
| Yes | 520 (27) | 236 (18) | 273 (18) |
| No | 1388 (72) | 1098 (82) | 1272 (82) |
| Unknown | 11 (1) | 1 (0.0) | 1 (0.0) |
| Retained |  |  |  |
| Yes | 1784 (93) | 1265 (95) | 1477 (96) |
| No | 135 (7) | 70 (5) | 69 (4) |
