# Supplementary material for: Economic evaluation of a cluster randomized, non-inferiority trial of differentiated service delivery models of HIV treatment in Zimbabwe
Source: PLOS Glob Public Health. 2023 Mar 13;3(3):e0000493. doi: 10.1371/journal.pgph.0000493 (PMC10021451; doi:10.1371/journal.pgph.0000493)
Supplement: S2 Table — (DOCX) [file pgph.0000493.s002.docx]

**S2 Table.** Unit costs

| **Cost categories** | **Cost** |
| --- | --- |
| Facility visit | $4.62 |
| CAG/CARG interaction | $1.88 |
| Community distribution visit | - |
| Viral load test | $14.42** |
| Monthly cost of first-line ART | $13.81** |

**Mavhu et al. Lancet Global Health 2020
